# Allostatic Load and Oxidative Stress Markers in Women With Early- and Late-Onset Preeclampsia

**DOI:** 10.7759/cureus.76516

**Published:** 2024-12-28

**Authors:** Araceli Montoya-Estrada, María Valeria García-Cerda, Alberto Martin Guzmán-Grenfell, Ricardo Figueroa-Damian, José Luis Torres-Cosme, Estibalitz Laresgoiti-Servitje, Jesús Jorge Beltrán-Montoya, José Romo-Yañez, Enrique Reyes-Muñoz

**Affiliations:** 1 Coordination of Gynecological and Perinatal Endocrinology, National Institute of Perinatology Isidro Espinosa de los Reyes, Mexico City, MEX; 2 Department of Immunobiochemistry, National Institute of Perinatology Isidro Espinosa de los Reyes, Mexico City, MEX; 3 Department of Infectology, National Institute of Perinatology Isidro Espinosa de los Reyes, Mexico City, MEX; 4 Research Division, Community Interventions, National Institute of Perinatology Isidro Espinosa de los Reyes, Mexico City, MEX; 5 Research Division, National Institute of Perinatology Isidro Espinosa de los Reyes, Mexico City, MEX; 6 Department of Obstetrics, National Institute of Perinatology Isidro Espinosa de los Reyes, Mexico City, MEX

**Keywords:** allostatic load, arginase, early-onset preeclampsia, late-onset preeclampsia, oxidative stress

## Abstract

Background: Allostatic load and oxidative stress (OS) markers differ in women with and without preeclampsia. However, there is no difference in allostatic load and OS markers between late-onset preeclampsia (L-OP) and early-onset preeclampsia (E-OP). This study aimed to compare the concentrations of allostatic load and OS markers in pregnant women with L-OP and E-OP.

Methods: This was a comparative cross-sectional study. Women with single pregnancy and a diagnosis of preeclampsia were included. Women with multiple pregnancies and/or chronic hypertension were excluded. Group 1 included women with E-OP (n=16), Group 2 included women with L-OP (n=45), and Group 3 included women without preeclampsia (n=40).

Results: No statistical differences were found in maternal age, body mass index, or number of gestations. Arginase levels were higher in Group 1 compared to Group 2 (p = 0.04) and Group 1 compared to Group 3 (p = 0.01). Total antioxidative capacity was higher in Group 1 compared to Group 2 (p = 0.03) and Group 1 compared to Group 3 (p = 0.001). Malondialdehyde levels were higher in Group 1 compared to Group 2 (p = 0.001) and Group 1 compared to Group 3 (p = 0.001). Total sulfhydryl compounds were lower in Group 1 compared to Group 2 (p = 0.01) and Group 1 compared to Group 3 (p = 0.001). No differences were observed between study groups in allostatic load markers.

Conclusions: Higher levels of OS markers were found in women with E-OP compared to women with L-OP and controls.

## Introduction

Hypertensive disorders of pregnancy are one of the leading causes of maternal and fetal morbidity and mortality worldwide, and preeclampsia complicates an estimated 2%-8% of pregnancies globally [1,2]. Preeclampsia corresponds to up to 70% of hypertensive disorders during pregnancy, of which 5%-20% occur before 34 weeks of gestation; this is known as early-onset preeclampsia (E-OP) [3]. Preeclampsia is characterized by hypertension and proteinuria after 20 weeks of gestation [1,4]. Risk factors include obesity, diabetes mellitus, preexisting chronic hypertension, low socioeconomic status, and maternal age, among others [5,6].

In Latin America and the Caribbean, hypertensive disorders are responsible for almost 26% of maternal deaths [1]. The pathophysiology of preeclampsia includes uterus-placental hypoperfusion coupled with generalized vasospasm, hypovolemia, and hypoperfusion of the maternal organs [2].

Currently, the investigation into the role of oxidative stress (OS) as a possible trigger of preeclampsia has gained attention. Based on the damage theory, placental endothelial and immune responses are released [2,6]. Several studies suggest that E-OP and late-onset preeclampsia (L-OP) may have distinct pathophysiological mechanisms. Alterations have been observed in the mechanisms of oxygen detection in E-OP but not in L-OP. Furthermore, E-OP, but not L-OP, is associated with increased placental OS and a higher plasminogen activator inhibitor (PAI) type 1 to PAI-2 ratio, supporting different pathophysiological mechanisms [7,8].

OS in the syncytiotrophoblast is a critical feature of preeclampsia. While normal placentation involves low oxygen tension followed by maternal blood flow oxygenation, intermittent hypoxia and reoxygenation caused by poor spiral artery invasion may induce OS [8]. Previous studies report increased placental production of reactive oxygen species (ROS), reactive nitrogen species, and lower levels of antioxidants in women with preeclampsia [3]. Preeclampsia has been associated with reduced superoxide dismutase and catalase activities and increased lipid peroxidation by-products in blood plasma [3]. Recently, increased ROS has been demonstrated in endothelial cells of the umbilical cord vein in women with preeclampsia [8].

The term "allostatic load" refers to the cumulative physiological burden resulting from adaptive responses to stressful stimuli, leading to wear and tear of regulatory systems and promoting biological dysregulation across various levels [9]. Allostatic load is commonly used to measure accumulated psychological stress through interactions with biomarkers of neuroendocrine, inflammatory, metabolic, and cardiovascular functions [9]. High allostatic load has been associated with adverse health outcomes, including adverse perinatal outcomes [5]. The primary mediators of allostatic load include cortisol, epinephrine, and norepinephrine [10]. Components of the hypothalamic-pituitary-adrenal axis, the sympathetic-adrenal-medullary axis, and the immune system are valuable markers for assessing the effects of stress and allostatic load [9,11]. Cortisol is a biomarker that can be positively or negatively regulated by stress [12-15]. Other biomarkers that increase in stressful situations include IL-6, urinary norepinephrine, dehydroepiandrosterone sulfate (DHEA-S), cholesterol, glycated hemoglobin, and triglycerides [14]. In addition to allostatic load, OS has been implicated in preeclampsia. The predisposition to OS and lipid peroxidation in women with preeclampsia may contribute to the presence of numerous extracellular neutrophil traps in the intervillous space of the placenta [16].

Few studies have compared biomarkers of allostatic load and OS in women with E-OP versus L-OP [3,6,11,14]. Therefore, the present study aims to compare the concentrations of OS and allostatic load markers in pregnant women with E-OP, L-OP, and a control group without preeclampsia.

## Materials and methods

Study design and population

A comparative cross-sectional study was conducted at the National Institute of Perinatology between December 2014 and March 2017. The protocol was approved by the institute’s Research, Ethics, and Biosafety Committees (register number 212250-34020102131) on June 12, 2014. All participants provided written informed consent.

The inclusion criteria encompassed women with a single pregnancy complicated by E-OP or L-OP, as well as a control group of women without preeclampsia. Participants were aged 18 to 40 years.

Exclusion criteria included multiple pregnancies, pregestational diabetes mellitus, and/or any of the following conditions: hyperthyroidism, systemic lupus erythematosus, rheumatoid arthritis, heart disease, chronic hypertension, kidney disease, liver disease, or cardiovascular diseases. Women who declined to participate in the study were also excluded.

Sample collection and analysis

Women were invited to participate when the diagnosis of preeclampsia was established. Each participant signed the informed consent form, and a blood sample was taken to measure the biomarkers of allostatic load and OS. Two tubes with whole blood samples were obtained after fasting for 8 to 12 hours from an antecubital venipuncture: one anticoagulated tube with 10.8 mg ethylenediaminetetraacetic acid (BD Vacutainer, Franklin Lakes, New Jersey) and one yellow tube with clotting agents and gel to separate the serum. The samples were centrifuged at 3,000 rpm for 15 minutes to obtain the plasma and serum and were stored in aliquots at −70°C until their analysis.

The concentration of cortisol, DHEA-S, glucose, total cholesterol, high-density lipoprotein (HDL), low-density lipoprotein (LDL), very-low-density lipoprotein (VLDL), triglycerides, uric acid, arginase, nitrites/nitrates, total antioxidant capacity, poly-o-aminophenols, tyrosines, and total sulfhydryls was measured in all participants.

The selected markers of allostatic load were chosen because they are practical in clinical settings. The OS markers were selected because they have well-established analytical techniques, allowing the collection of robust and reproducible data to validate the study. They are also concrete indicators of oxidative damage to lipids and proteins, meaning that their quantification can accurately reflect the impact of oxidative stress on the evaluated samples.

Study variables

The study’s primary objective was to compare the concentration of OS and allostatic load markers among women with E-OP, L-OP, and control women without preeclampsia. Preeclampsia was defined as systolic blood pressure ≥140 mmHg or diastolic blood pressure ≥90 mmHg on two occasions at least 4 hours apart, accompanied by proteinuria (≥300 mg per 24-hour urine collection or dipstick reading ≥1+) after 20 weeks of gestation in a woman with previously normal blood pressure. In the absence of proteinuria, preeclampsia was diagnosed based on the presence of thrombocytopenia (<100,000/µL), creatinine >1.1 mg/dL, elevated transaminases (≥2 times the normal value), pulmonary edema, or cerebral/visual symptoms [1]. E-OP was defined as preeclampsia diagnosed before 34 weeks of gestation, while L-OP was defined as preeclampsia diagnosed at or after 34 weeks of gestation.

OS marker variables included arginase, nitrates, total antioxidant capacity, advanced oxidation protein products (AOPPs), dityrosines, total sulfhydryls, carbonyls, lipohydroperoxides, malondialdehyde (MDA), and paraoxanase.

Clinical records provided the following variables: maternal age, marital status, number of previous pregnancies, weight and height at the beginning of pregnancy, pre-pregnancy body mass index (BMI), socioeconomic status, education level, associated comorbidities, history of infertility, family history of type 2 diabetes mellitus, chronic hypertension, obesity, acetylsalicylic acid intake during pregnancy, severity of preeclampsia, and perinatal outcomes. Additional data collected at the time of preeclampsia diagnosis included gestational age, systolic and diastolic blood pressure, proteinuria (Labstix), hemoglobin, hematocrit, leukocytes, platelets, serum creatinine, urea, uric acid, direct bilirubin, indirect bilirubin, aspartate aminotransferase, alanine aminotransferase, and lactic dehydrogenase. Perinatal outcomes included gestational age at delivery, maternal weight and BMI at delivery, mode of delivery, and length of maternal hospital stay. Neonatal variables included length of hospital stay, birth weight, birth height, neonatal outcomes at delivery, and congenital malformations.

Procedure 

Arginase activity was evaluated by measuring the release of urea from L-arginine using Corraliza’s method [17], with absorbance measured spectrophotometrically at 544 nm. Nitrites and nitrates were measured according to the method of Miranda et al. [18], which involves the reduction of the nitrate anion to nitrite using vanadium chloride, followed by spectrophotometric detection of nitrite through the Griess reaction.

Total antioxidant activity was determined using the CUPRAC method as described by Apak et al. [19]. This method measures cupric-reducing antioxidant capacity using copper (II) and neocuproine reagents. A spectrophotometric assay was performed at 450 nm, with Trolox used as the water-soluble analogue of vitamin E.

AOPPs, representing structural alterations in proteins caused by oxidative stress (OS), were measured using the method of Hanasand et al. [20], with absorbance read at 340 nm. AOPP concentrations were expressed in mM.

Dityrosine was measured by fluorescence following the method of Malencik et al. [21]. Fifty microliters of the sample was mixed with 1,950 μL of 6 M urea-0.1 M sodium bicarbonate at pH 9.8. After 30 minutes at ambient temperature, fluorescence was measured using a Turner Designs TD-700 fluorometer, previously calibrated with one μM quinine sulfate. Excitation was set at 320 nm and emission at 405 nm. The dityrosine concentration was calculated with a standard curve realized with dityrosine synthesized according to Malencik et al. [21] and expressed as nmol/mg protein.

Protein and non-protein sulfhydryls in erythrocytes were quantified spectrophotometrically using Ellman’s reagent [22]. Carbonyl content in plasma was determined using 2,4-dinitrophenyl hydrazine, which reacts with carbonyl groups to form stable hydrazones. These were measured spectrophotometrically at 370 nm, as described by Dalle Donne et al. [23], and expressed as pmol PC/mg protein.

Lipohydroperoxides were measured spectrophotometrically at 360 nm following the assay conditions outlined by El-Saadani [24]. MDA, a key end-product of lipid peroxidation and a representative marker of oxidative lipid damage, was quantified using 1-methyl-2-phenylindole with a spectrophotometric assay at 586 nm [25].

Paraoxonase activity was assessed by measuring the hydrolysis of diethyl p-nitrophenyl phosphate (paraoxon), with absorbance recorded at 405 nm. Enzyme activity was calculated using the molar extinction coefficient of p-nitrophenol (18,053 (mol/L)^−1^ cm^−1^) [26].

The allostatic load markers were measured in serum as follows: cortisol, insulin, and DHEA-S levels were measured by chemiluminescence (Immulite 2000, Siemen’s Healthcare Diagnostics Inc., Deerfield, Illinois) considering insulin sensitivity: 2 μIU/mL and coefficient of variation (CV): 4.1%-7.3%, DHEA-S: 3 μg/mL, CV: 9.3%-13.0%, and cortisol sensitivity: 0.2 μg/mL, CV: 5.8-7.8%.

Sample size

Because few studies have compared allostatic load and OS biomarkers in women with E-OP versus L-OP, a sample size was calculated considering a power of 80%, an alpha of 0.05, and a mean effect size of 0.20 in the difference in means of the biomarkers analyzed between the L-OP and E-OP. With a minimal sample size of 15 participants per group, we included all participants who fulfilled the inclusion criteria during the study period. 

Statistic analysis

Qualitative variables were described with frequencies and percentages, and quantitative variables with mean and standard deviation. Each allostatic load and OS marker was compared through a one-way ANOVA or Kruskal-Wallis test, according to the distribution of the variable. A value of p < 0.05 was considered significant. Statistical analysis was performed using IBM SPSS Statistics for Windows, Version 24 (Released 2016; IBM Corp., Armonk, New York).

## Results

During the study period, a total of 101 participants were included: Group 1 consisted of 16 women with E-OP, Group 2 included 45 women with L-OP, and Group 3 comprised 40 control women without preeclampsia. Table 1 presents the general characteristics of the participants. A statistically significant difference was observed in acetylsalicylic acid intake during pregnancy between Group 1 (E-OP) and the control group (Group 3); no other significant differences were noted among the groups at study admission.

**Table 1:** Maternal characteristics at admission to the study for early-onset preeclampsia, late-onset preeclampsia, and control groups. E-OP: early-onset preeclampsia, L-OP: late-onset preeclampsia, p-BMI: pre-gestational body mass index (kg/m^2^).

Maternal characteristics at admission	E-OP, n=16	L-OP, n=45	p, E-OP versus L-OP	Controls without preeclampsia, n=40	p, E-OP versus control	p, L-OP versus control
Age (years)	31.0 ± 6.2	30.0 ± 7.7	0.98	28.7± 8.9	0.98	0.98
Parity	2.2 ± 1.3	2.4 ± 1.5	0.97	2.4 ± 1.5	0.98	0.98
Primipara	7 (43.8%)	16 (35.6%)	0.78	16 (40%)	0.96	0.84
Two or more pregnancies	9 (56.2%)	29 (64.4%)	0.78	24 (60%)	0.96	0.84
Height (m)	1.59 ± 0.06	1.55 ±0.06	0.95	1.59 ±0.63	0.97	0.01
Pregestational weight	70.8 ±17.9	65.0 ±14.8	0.52	69.5 ±13.1	0.97	0.46
Normal weight (p-BMI = 18.5-24.99 kg/m^2^)	7 (43.8%)	21 (46.7%)	0.92	16 (40%)	0.96	0.69
Overweight (p-BMI = 25-29.9 kg/m^2^)	3 (18.8%)	15 (33.3%)	0.43	17 (42.5%)	0.17	0.51
Obesity (p-BMI >30 kg/m^2^)	6 (37.4%)	9 (20%)	0.29	7 (17.5%)	0.21	0.98
Preeclampsia with severity data	7 (43.8%)	21 (46.7%)	0.92	0 (0%)	-	-
Acetylsalicylic acid intake during pregnancy	10 (62.5%)	16 (35.6%)	0.11	9 (22.5%)	0.01	0.28

Table 2 shows the personal and family history of the women among groups of the study. There were no statistically significant differences in history of infertility, diabetes, obesity, and systemic hypertension.

**Table 2:** Personal and Family history of the women at admission to the study for early-onset preeclampsia, late-onset preeclampsia, and control groups. E-OP: early-onset preeclampsia, L-OP: late-onset preeclampsia.

Maternal history	E-OP, n=16	L-OP, n=45	p, E-OP versus L-OP	Controls without preeclampsia, n=40	p, E-OP versus control	p, L-OP versus control
History of infertility	5 (31.3%)	8 (17.8%)	0.43	4 (10%)	0.12	0.47
First-degree family history of diabetes	12 (75%)	31 (68.9%)	0.88	22 (55%)	0.27	0.27
Family history of obesity	7 (43.8%)	14 (31.1%)	0.54	17 (42.5%)	0.83	0.38
Family history of chronic hypertension	9 (56.3%)	19 (42.2%)	0.50	19 (47.5%)	0.76	0.78

The clinical and laboratory characteristics at admission to the study for women with E-OP, L-OP, and the control group without preeclampsia are presented in Table 3. There were no significant differences in clinical and laboratory characteristics between the E-OP and L-OP groups, except for the gestational age at the time of preeclampsia diagnosis. As expected, systolic and diastolic blood pressure, proteinuria, leukocyte counts, and uric acid levels were significantly higher in both preeclampsia groups compared to the control group without preeclampsia.

**Table 3:** Clinical and laboratory characteristics at admission to the study among women with early-onset and late-onset preeclampsia and the control group without preeclampsia. E-OP: early-onset preeclampsia, L-OP: late-onset preeclampsia, NA: not applicable, AST: aspartate aminotransferase, ALT: alanine aminotransferase, LDH: lactate dehydrogenase.

Preeclampsia laboratories	E-OP, n=16	L-OP, n=45	p, E-OP versus L-OP	Controls without preeclampsia, n=40	p, E-OP versus control	p, L-OP versus control
Weeks of gestation at diagnosis of preeclampsia	27.1 ± 4.9	37.0 ±2.1	0.001	NA	-	-
Systolic blood pressure at diagnosis (mmHg)	152.2 ± 19.2	149.2 ± 14.2	0.98	110.5 ± 9.4	0.001	0.001
Diastolic blood pressure at diagnosis (mmHg)	95.1 ± 9.4	94.4 ±8.7	0.97	75.3 ± 6.3	0.005	0.001
Proteinuria by labstix (+)	1.6 ± 1.0	1.7 ± 1.0	0.97	0.0 ± 0	0.001	0.001
Hemoglobin (g/dL)	12.7 ±1.1	12.6 ± 2.3	0.98	12.5 ±1.4	0.96	0.97
Hematocrit (%)	35.8 ± 3.8	38.3 ± 4.3	0.95	38.0 ± 3.7	0.21	097
Leukocytes (n/mm^3^)	10,150 ± 2,031	8,835 ± 2,731	0.59	4,446 ± 4,658	0.001	0.001
Platelets (10^3^/mcL)	223.1 ± 76.6	207.0 ±70.0	0.98	231.1 ± 45.8	0.97	0.27
Serum creatinine (mg/dL)	0.5 ± 0.2	0.6 ± 0.3	0.31	0.5 ± 0.1	0.98	0.11
Urea (mg/dL)	21.0 ± 10.0	20.7 ± 12.7	0.97	15.2 ± 4.7	0.20	0.07
Uric acid (mg/dL)	4.9 ± 1.7	5.5 ± 1.4	0.26	3.8 ± 1.0	0.05	0.001
Direct bilirubin (mg/dL)	0.1 ± 0.07	0.2 ± 0.2	0.97	0.1 ± 2.0	0.96	0.98
Indirect bilirubin (mg/dL)	0.6 ± 1.8	0.2 ± 0.4	0.45	0.1 ± 0.1	0.98	0.97
AST (IU/L)	19.0 ± 6.8	20.4 ± 8.3	0.98	21 ±10.8	0.98	0.97
ALT (IU/L)	19.3 ± 10.7	16.5 ± 6.6	0.69	26.8 ± 11.1	0.31	0.05
DHL (IU/L)	325.6 ± 83.4	445.9 ± 820.4	0.96	389.7±147.4	0.97	0.98

Table 4 shows the oxidative stress markers. An association was found between E-OP (Group 1) and L-OP (Group 2). The following studied markers showed statistical significance: arginase, total antioxidant capacity, total sulfhydryls, and malondialdehyde. Arginase levels increased by 61% in Group 1 compared to the control group. Total antioxidant capacity was statistically significantly increased, being 1.8 times higher in Group 1 and 1.5 times higher in Group 2 compared to the control group. Conversely, total sulfhydryls decreased compared to the control group.

**Table 4:** Oxidative stress biomarkers among women with early-onset and late-onset preeclampsia and the control group without preeclampsia. E-OP: early-onset preeclampsia, L-OP: late-onset preeclampsia, AOPPs: advanced oxidation protein products.

Biomarkers of oxidative stress	E-OP, n=16	L-OP, n=45	p, E-OP versus L-OP	Controls without preeclampsia, n=40	p, E-OP versus control	p, L-OP versus control
Arginase (nmol/min/mg)	68.0 ± 30.7	40.5 ± 14.9	0.04	42.1 ± 12.9	0.01	0.98
Nitrites/nitrates (nmol/mL)	36.4 ± 8.2	31.0 ± 8.2	0.59	33.9 ± 12.3	0.97	0.98
Total antioxidant capacity (µmol eq trolox/mL)	5.8 ± 1.2	4.9 ± 1.1	0.03	3.1 ± 1.0	0.001	0.001
AOPPs (µM)	187.2 ± 63.2	197.0 ± 87.0	0.97	115.6 ± 53.9	0.07	0.001
Dityrosines (µM)	301.9 ± 124.3	347.4 ± 65.1	0.44	295.1 ± 89.0	0.97	0.13
Total sulfhydryls (nmol/mL)	287.3 ± 64.0	335.0 ± 48.5	0.01	387.4 ± 34.3	0.001	0.001
Carbonyls (nmoles PC/mg protein)	5.2 ± 3.1	4.3 ± 3.0	0.97	7.3 ± 2.1	0.09	0.001
Lipohydroperoxides (nmoles/mg dry weight)	0.03 ± 0.01	0.04 ± 0.01	0.50	0.03 ± 0.1	0.98	0.02
Malondialdehyde (nmoles MDA/mg dry weight)	0.1 ± 0.1	0.06 ± 0.07	0.001	0.04 ± 0.01	0.001	0.70
Paraoxanase (nmoles/mg protein/min)	0.1 ± 0.06	0.2 ± 0.06	0.97	0.2 ± 0.13	0.98	0.96

Allostatic load markers, referring to DHEA-S, serum cortisol, glucose, cholesterol, triglycerides, HDL, LDL, and VLDL, did not show any association among the three study groups. The results of allostatic load markers are shown in Table 5.

The association between oxidative stress biomarkers, with a predominance in E-OP and L-OP, was observed, compared with the null association observed in the results for allostatic load markers.

The comparison of allostatic load markers between E-OP and L-OP groups did not show significant differences; however, the mean levels of DHEA-S, serum cortisol, and insulin were lower in E-OP. The lack of substantial differences in allostatic load markers could be attributable to the sample size in the E-OP group and the wide distribution of the data. Compared to E-OP versus the control group, serum cortisol was significantly lower, and triglycerides were significantly higher in the E-OP group. There were no other differences among the three groups. The results of allostatic load markers are shown in Table 5.

**Table 5:** Allostatic load markers among early-onset and late-onset preeclampsia and women without preeclampsia. E-OP: early-onset preeclampsia, L-OP: late-onset preeclampsia, DHEA-S: dehydroepiandosterone sulfate, HDL: high-density lipoprotein, LDL: low-density lipoprotein, VLDL: very low-density lipoprotein.

Allostatic load markers	E-OP, n=16	L-OP, n=45	p, E-OP versus L-OP	Controls without preeclampsia, n=40	p, E-OP versus control	p, L-OP versus control
DHEA-S (µg/dL)	35.2 ± 29.0	52.4 ± 40.5	0.53	44.5 ± 40.6	0.98	0.97
Serum cortisol (nmol/L)	13.2 ± 12.5	18.0 ± 7.3	0.21	20.5 ± 7.3	0.02	0.56
Insulin (uU/L)	18.1 ± 29.9	20.8 ± 20.1	0.98	27.5 ± 32.0	0.86	0.84
Glucose (mg/dL)	85.8 ± 27.9	83.1 ± 32.1	0.97	86.5 ± 37.2	0.97	0.96
Cholesterol (mg/dL)	217.7 ± 67.0	212.9 ± 73.3	0.96	227.6± 86.3	0.96	0.98
Triglycerides (mg/dL)	374.0 ± 134.0	354.2 ± 137.9	0.97	279.0± 87.4	0.04	0.02
HDL (mg/dL)	65.7 ± 17.0	63.2 ± 19.3	0.97	60.3 ± 18.2	0.97	0.96
LDL (mg/dL)	93.5 ± 58.3	90.8 ± 59.4	0.98	114.5± 65.8	0.98	0.45
VLDL (mg/dL)	65.0 ±16.6	61.1 ±18.3	0.96	56.3 ± 17.3	0.39	0.70

Table 6 shows pregnancy resolution outcomes among women with E-OP, L-OP, and the control group without preeclampsia. Weeks at resolution, newborn weight, and height were significantly lower in E-OP compared to L-OP. Maternal length of stay and admission to the neonatal intensive care unit were higher in E-OP compared to L-OP.

**Table 6:** Pregnancy resolution outcomes among women with early-onset and late-onset preeclampsia and the control group without preeclampsia. E-OP: early-onset preeclampsia, L-OP: late-onset preeclampsia, NICU: neonatal intensive care unit, INICU: intermediate neonatal intensive care unit.

Perinatal outcomes	E-OP, n=16	L-OP, n=45	p, E-OP versus L-OP	Controls without preeclampsia, n=40	p, E-OP versus control	p, L-OP versus control
Weeks of gestation to resolution	31.1 ± 3.3	37.9 ± 2.2	0.001	39.2 ± 1.2	0.001	0.001
Vaginal, n (%)	0 (0%)	7 (15.6%)	0.32	20 (50%)	0.002	0.002
Cesarean section, n (%)	16 (100%)	38 (84.4%)	0.32	20 (50%)	0.002	0.002
Maternal length of stay (days)	5.3 ± 2.7	2.9 ± 0.8	0.001	2.6 ± 0.8	0.001	0.98
Newborn length of stay of (days)	4.5 ± 2.5	3.5 ± 4.4	0.87	2.85 ± 1.2	0.26	0.98
Newborn weight (g)	1,292.4 ± 411.9	2,633.9 ± 615.3	0.001	3,213.6 ± 390.3	0.001	0.001
Newborn height (cm)	39.4 ± 4.3	47.4 ± 3.5	0.001	50.0 ± 1.6	0.001	0.01
INICU, n (%)	3 (20%)	9 (20%)	0.79	8 (20%)	0.79	0.78
NICU, n (%)	6 (40%)	3 (6.7%)	0.01	1 (2.5%)	0.002	0.69
Neonatal malformations	1 (6.7%)	1 (2.2%)	0.96	0(0%)	0.96	0.96

## Discussion

In the present study, we found that women with E-OP have a higher concentration of markers of OS compared to women with L-OP or women without preeclampsia. Regarding allostatic load biomarkers, there were no differences in the concentration of glucose, insulin, DHEA-S, cholesterol, HDL, LDL, and VLDL, except for triglycerides and cortisol. This could be partly explained by the limited sample size and the fact that the control group was pregnant at term.

Women with E-OP had higher acetylsalicylic acid intake during pregnancy than those with L-OP. This result could be attributed to the fact that, according to the institutional protocol of antenatal care, women at high risk of developing preeclampsia (based on clinical history and/or Doppler of uterine arteries) were recommended prophylaxis with acetylsalicylic acid before 16 weeks of gestation in all three groups. A significant difference was observed between E-OP and the control group (62.5% vs. 22.5%). No relevant findings emerged when assessing the women’s hereditary family history.

When analyzing the results of initial laboratory tests for the diagnosis of preeclampsia, a significant difference was found in leukocyte counts among women with E-OP and L-OP compared to the control group. Similar results were observed in a study of OS by León-Reyes et al. [27], who reported an elevation in uric acid of up to 30% in women with preeclampsia. The same results were observed in women with both E-OP and L-OP compared to the control group, without other laboratory tests showing statistical significance.

The OS markers provided favorable results for the study, demonstrating their increase in women with both E-OP and L-OP. Paraoxonase activity was reduced in up to 59% of women with preeclampsia in the study by León-Reyes et al. [27], while no significant differences were observed in the current study. These differences may be due to the fact that León-Reyes et al. [27] evaluated lipoprotein particles (LDL-c and HDL-c), whereas the present study evaluated plasma samples. Similarly, studies by Poston et al. [28] and Williamson et al. [29] reported a slight increase in MDA, one of the most specific OS markers, in women with preeclampsia compared to the control group, consistent with our findings.

This study found significant results in arginase, total antioxidant capacity, total sulfhydryls, and MDA among the OS biomarkers. These markers could currently serve as potential indicators for ruling out preeclampsia in suspected cases. However, further research is needed to identify more specific laboratory tests for diagnosing preeclampsia.

In their study of 117 women, Hux and Roberts compared women with preeclampsia to a control group and found a higher allostatic load in women with preeclampsia compared to the control group [6]. This analysis suggests an absence of multisystemic subclinical regulation associated with preeclampsia. However, the current study did not observe these results. On the contrary, a decrease in LDL (without statistical significance) was observed in women with preeclampsia versus the control group, contrasting with the results reported by León-Reyes et al. [27], who noted a slight increase in LDL among women with preeclampsia. This discrepancy in findings could be attributed to differences in sample size, diet, and data distribution.

Vianna et al. [30] reported that altered cortisol metabolism could trigger an increase in insulin resistance and serum cortisol during pregnancy due to hypertension and endothelial damage. However, when analyzing the allostatic load marker results table, the opposite was reported in a study by Vianna et al., who found a decrease in the presence of serum cortisol in women with preeclampsia versus the control group: Group 1 versus Group 3 (13.2 ± 12.5 vs. 20.5 ± 7.3) and Group 2 versus Group 3 (18 ± 17.3 vs. 20.5 ± 7.3). This could be associated with a chronic stress event rather than an acute event, in which cortisol levels would typically be elevated, as described by Vianna et al. [30].

We did not find clinically significant results when analyzing the lipid profile, insulin, cortisol, and DHEA-S in women with E-OP and L-OP. However, it is worth highlighting the significant alteration in triglycerides in both preeclampsia groups compared to the control group.

Finally, within the perinatal results, we found that the resolution route in women with E-OP was the abdominal route. In contrast, for women with L-OP, more than two-thirds of the pregnancies were resolved by the abdominal route and the remainder by the vaginal route, similar to those reported by van der Merwe [11]. For the control group, 50% were resolved vaginally, and the other 50% abdominally, different from the study of Leon-Reyes et al. [27], who reported delivery by cesarean section in 100% of the control group. Likewise, there was a greater need for neonatal admission to intensive care for E-OP, with statistical significance. However, it should be emphasized that these were preterm births, which is why the increase in the incidence of neonatal therapy was justified compared to the L-OP group, which was end products. The mean gestational age at which women with E-OP resolved was 31.1 weeks, six weeks earlier than women with L-OP, who delivered at 38 weeks gestation. It should be noted that the average hospital stay for E-OP patients was longer than the stay of women with L-OP and the control group, similar to those reported in previous studies [5].

In the present study, allostatic load markers were not significantly different among the three groups, except for triglycerides and cortisol. This finding could be partly attributed to the limited sample size and the fact that the control group consisted of term pregnancies.

The main limitations of this study include its design and the small sample size in the E-OP group. Additionally, being a single-center study constrains the generalizability of the results.

A strength of this study lies in its combined evaluation of OS and allostatic load markers in a clinical setting, focusing on Latin American women with E-OP and L-OP, a population with limited reported data.

Subsequent research should be conducted with a large sample size, a multicenter design, and different body mass index groups to reach a more definitive conclusion. On the other hand, the efficacy of antioxidants in preventing preeclampsia is controversial [4]. Likewise, large, multicenter clinical trials are necessary to evaluate the effect of antioxidants, particularly L-arginine/L-citrulline supplementation, on preventing preeclampsia in Latin American and non-Latin American women. 

## Conclusions

An association was found with specific biomarkers of OS for women with E-OP and L-OP; however, no association with allostatic load markers was found. Statistical significance was noted for four OS markers: arginase, total antioxidant capacity, total sulfhydryls, and MDA; their use in the future as diagnostic markers for preeclampsia could be evaluated. Likewise, the potential use of antioxidants to prevent preeclampsia should be studied in the Latin American population.
